# Prognostic Risk Model of Immune-Related Genes in Colorectal Cancer

**DOI:** 10.3389/fgene.2021.619611

**Published:** 2021-03-04

**Authors:** Yucheng Qian, Jingsun Wei, Wei Lu, Fangfang Sun, Maxwell Hwang, Kai Jiang, Dongliang Fu, Xinyi Zhou, Xiangxing Kong, Yingshuang Zhu, Qian Xiao, Yeting Hu, Kefeng Ding

**Affiliations:** ^1^Department of Colorectal Surgery and Oncology, Key Laboratory of Cancer Prevention and Intervention, Ministry of Education, The Second Affiliated Hospital, Zhejiang University School of Medicine, Hangzhou, China; ^2^Zhejiang University Cancer Center, Zhejiang University, Hangzhou, China

**Keywords:** colorectal cancer, immune-related gene, immune prognostic signature, TCGA, tumor immune microenvironment

## Abstract

**Purpose:**

We focused on immune-related genes (IRGs) derived from transcriptomic studies, which had the potential to stratify patients’ prognosis and to establish a risk assessment model in colorectal cancer.

**Summary:**

This article examined our understanding of the molecular pathways associated with intratumoral immune response, which represented a critical step for the implementation of stratification strategies toward the development of personalized immunotherapy of colorectal cancer. More and more evidence shows that IRGs play an important role in tumors. We have used data analysis to screen and identify immune-related molecular biomarkers of colon cancer. We selected 18 immune-related prognostic genes and established models to assess prognostic risks of patients, which can provide recommendations for clinical treatment and follow-up. Colorectal cancer (CRC) is a leading cause of cancer-related death in human. Several studies have investigated whether IRGs and tumor immune microenvironment (TIME) could be indicators of CRC prognoses. This study aimed to develop an improved prognostic signature for CRC based on IRGs to predict overall survival (OS) and provide new therapeutic targets for CRC treatment. Based on the screened IRGs, the Cox regression model was used to build a prediction model based on 18-IRG signature. Cox regression analysis revealed that the 18-IRG signature was an independent prognostic factor for OS in CRC patients. Then, we used the TIMER online database to explore the relationship between the risk scoring model and the infiltration of immune cells, and the results showed that the risk model can reflect the state of TIME to a certain extent. In short, an 18-IRG prognostic signature for predicting CRC patients’ survival was firmly established.

## Introduction

Colorectal cancer (CRC) ranks among the top causes of cancer-related deaths worldwide that endangers human health. The GLOBOCAN data in 2018 released by the International Cancer Research Agency showed that each year there were approximately 1.85 million new CRCs and more than 880,000 deaths worldwide. The morbidity and mortality of CRC rank third and second, respectively, in malignant tumors, in which the morbidity accounts for approximately 10% of the total cancer incidence, and the mortality accounts for 9% of the total deaths due to cancer (Bray et al., 2018). It was predicted that the number of cases will increase by more than 60% in 2030, with 2.2 million new cases and 1.1 million deaths (Arnold et al., 2017). Surgical resection is the main treatment option for CRC patients. With the application and popularity of colonoscopy, early treatment work has been improved. The clinical outcomes of CRC patients in many countries have improved significantly over the past few decades (Atkin et al., 2017). Despite the complete surgical resection, many CRC patients eventually relapsed and developed metastatic disease (Angenete, 2019). In clinical practice, a more effective prognostic evaluation system is urgently needed to provide personalized medicine for CRC patients and improve patient outcomes.

It is noteworthy that after Fearon and Vogelstein proposed the model of CRC genetic basis, researchers have begun to understand the heterogeneity of CRC (Fearon and Vogelstein, 1990). Patients with different genetic backgrounds had different outcomes after receiving the same treatment (Fearon and Vogelstein, 1990). Some researchers believed that it was attributed to immunity-related factors (Becht et al., 2016). As we knew that the immune system plays an important role in the development of a variety of cancers, including CRC (Gentles et al., 2015). A recent study found that immunological data (such as type, density, and location of immune cells in tumor samples) can predict patient survival better than the current histopathological characteristics used for CRC patients (Galon et al., 2006). Immune cells are important parts of the tumor microenvironment and affect the development and metastasis of CRC (Pages et al., 2005). Tumor-infiltrating macrophages and dendritic cells in CRC are related to local regulatory T cells and systemic T-cell responses to tumor-associated antigens and have an impact on patients’ survival (Nagorsen et al., 2007). In addition, studies have shown that immune-related genes (IRGs) in colon cancer are closely related to the occurrence and development of colon cancer. However, there is currently no prognostic model based on IRGs to predict the overall prognosis of CRC patients and systematically assess the immune environment of CRC (Ge et al., 2019). Therefore, constructing an immune-based prognostic model that can effectively predict the prognosis of CRC has a very important clinical application prospect.

In this study, we screened differentially expressed IRGs that are closely related to CRC through bioinformatics analysis of The Cancer Genome Atlas (TCGA). Next, the IRGs that were significantly associated with prognosis were further screened. Differentially expressed tumor-associated transcription factors (TFs) were searched, and a correlation network was constructed to reveal the relationship between TFs regulating immune genes. Then, immune-related prognostic models were constructed by integrating IRGs of CRC. Besides, we verified that the risk model can be used as an effective independent prognostic indicator.

## Materials and Methods

### Patient Data Collection

Colorectal cancer patients (adenocarcinomas) with gene expression profiles and clinical information were obtained from TCGA data portal^1^. Processed RNA-Seq FPKM data of 398 CRC and 39 adjacent normal tissues were downloaded for further analyses.

### IRGs and Cancer-Related Transcription Factors

The comprehensive list of IRGs was downloaded from the Immunology Database and Analysis Portal (ImmPort) database^2^, which shares immunology data and provides a list of IRGs for cancer researchers (Bhattacharya et al., 2014). The IRGs that actively participated in the immune process were identified. To investigate the regulatory mechanism of IRGs, we extracted cancer-related transcription factors (CRTFs) for subsequent research. The CRTF data were downloaded from the Cistrome Cancer database^3^, which is a useful database for biomedical and genetic research and includes 318 CRTFs (Mei et al., 2017).

### Differential Gene Expression Analysis

To select the IRGs and TFs that contributed to the development and progression of CRC, differentially expressed genes (DEGs) between tumor samples and normal samples were screened using the limma R package. Differential expression analysis was conducted, with an adjusted false discovery rate < 0.05 and | log2(fold change)| > 1 as the thresholds. Differentially expressed IRGs were identified as overlaps between the IRG list and the DEG list. Differentially expressed TFs were identified as overlaps between the TFs list and the DEG list. Heatmaps were generated using the “pheatmap” R package, and volcano plots were also displayed using the “ggplot2” R package.

### CRTF-IRG Regulatory Network

In order to evaluate how differentially expressed CRTFs regulate prognosis-related IRGs, we studied the correlation between them. The core method is the Pearson test. The critical standard is set to a correlation coefficient > 0.4, *P* < 0.001. This step is performed using the Cor. test function in R, and the correlation coefficient and *P* value are calculated by Cor. test. To make the situation clearer, Cytoscape was used to build a visual regulatory network.

### PPI Network Construction and Module Analysis

The PPI network was predicted using an online database search tool STRING^4^ (Franceschini et al., 2013). Analyzing functional interactions between proteins may provide insights into the mechanisms of CRC development and progression. In this study, prognostic-related PPI networks of IRGs and CRTFs were constructed using the STRING database, and interactions with composite scores > 0.4 were considered statistically significant. Kyoto Encyclopedia of Genes and Genomes signaling pathways and biological functions of genes were analyzed using functional clustering carried by STRING.

### Gene Set Enrichment Analysis

Gene Set Enrichment Analysis (GSEA)^5^ was used to analyze the GO term of the genes that make up the signature.

### Construction of the Immune-Related Signature for CRC

To control the quality of the data, after excluding patients who lacked survival information or survived for less than 90 days, 334 samples were subsequently analyzed. Transcriptomic analysis of RNA measured by FPKM values was performed using log2-based conversion. Based on the differentially expressed IRGs, Kaplan–Meier analysis was first performed to screen prognostic immune genes. Then, immune-related prognostic signature (IPS) was constructed by multivariate Cox regression to calculate the risk score for each patient. Risk scores were acquired based on expressions of genes multiplied by a linear combination of regression coefficients obtained from the multivariate Cox regression analysis. *P* < 0.01 was regarded as significant.

### Survival Analysis

According to the optimal cutoff value obtained by the “survminer” R package, CRC patients were classified into low risk and high risk according to their risk scores. To investigate the prognostic value of the prognostic model in CRC patients, univariate Cox analysis was implemented by the “survival” R package and “survminer” R package. A time-dependent receiver operating characteristic (ROC) curve was plotted to assess sensitivity and specificity using the “timeROC” R software package (Heagerty et al., 2000). The area under the curve was calculated from the ROC curve.

### Association Analysis Between 18-IRGs and Clinical Parameters

Association analysis of clinical characteristics of 18 key prognostic IRGs in the model was performed using the *t* test. To transform the data types into binary variables, 398 CRC patients were grouped according to different clinical characteristics. In terms of age, 65 years old was chosen as the cutoff point. The stage was divided into stages I and II and stages III and IV. The T stage was divided into T1–2 and T3–4. M stage was divided into M0 or M1. N stages N0 and N1–2.

### TIMER Database Analysis of the Correlation Between Immune-Related Markers and Immune Cell Infiltration

TIMER database^6^ is a comprehensive resource for systematical analysis of immune infiltrates across different cancer types (Li et al., 2017). The abundance of six immune infiltrates was estimated by the TIMER algorithm (B cells, CD4^+^ T cells, CD8^+^ T cells, neutrophils, macrophages, and dendritic cells). We used the TIMER database to analyze the correlation between the prognostic model of CRC patients and six tumor-infiltrating immune cells.

### Statistical Analysis

Overall survival (OS) was defined as the main outcome. Univariate cox regression analysis and multivariate cox regression analysis were performed to evaluate the prognostic effect of the immune signature and various clinicopathological features including age, clinical stage, grade, and TNM stage. Statistical analyses were performed using R software (version 3.5.1). The heatmap was generated using the “pheatmap” R package. Unless otherwise specified, a two-sided *P* < 0.05 was considered statistically significant.

## Results

### Differentially Expressed IRGs and CRTFs in CRC

**FIGURE 1:**
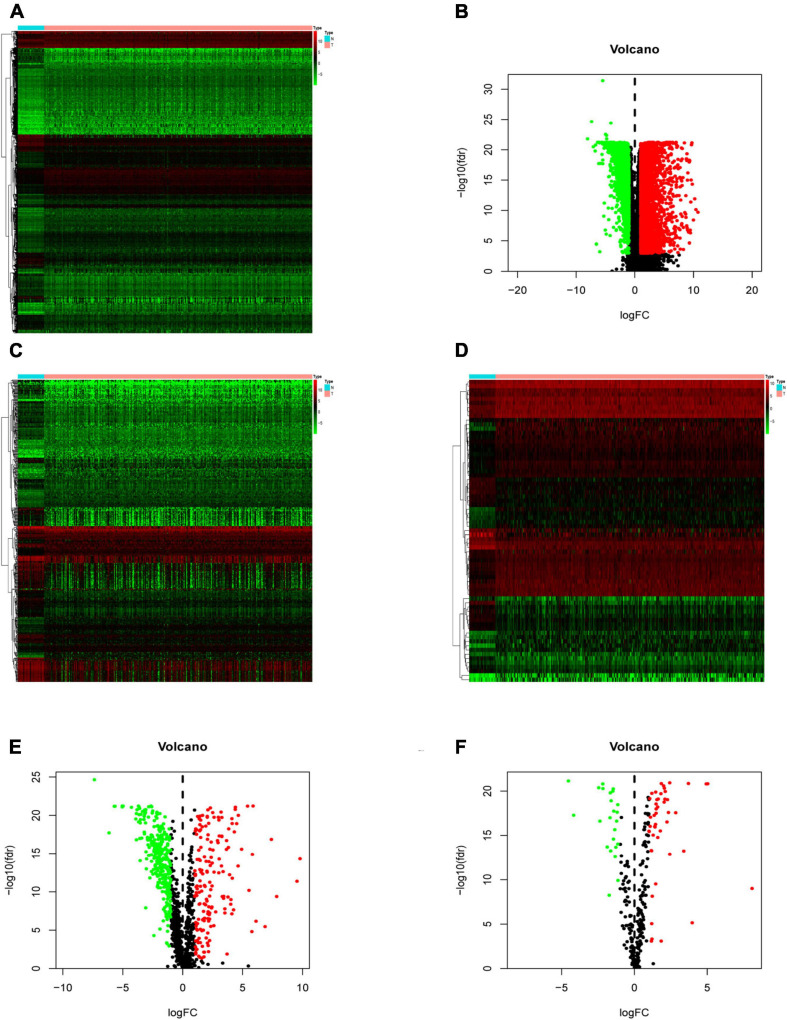
Differentially expressed immune-related genes (IRGs) and cancer-related transcription factors (CRTFs) in colorectal cancer (CRC). **(A)** Heatmap of differentially expressed genes in CRC. The color from green to red represents the progression from low expression to high expression. **(B)** Volcano plot of differentially expressed genes in CRC. The red dots in the plot represent upregulated genes, and green dots represent downregulated genes with statistical significance. Black dots represent no differentially expressed genes in CRC. **(C)** Heatmap of significantly differentially expressed IRGs in CRC. **(D)** Heatmap of significantly differentially expressed cancer-related transcription factors in CRC. The color from green to red represents the progression from low expression to high expression. **(E)** Volcano plot of differentially expressed IRGs. **(F)** Volcano plot of differentially expressed cancer-related transcription factors.

Compared with normal tissues, there were 5,938 DEGs in CRC tissues, of which 3,936 were up-regulated and 2,002 were down-regulated in these samples. The difference between tumor tissue and normal tissue can be seen through the heatmap and the volcano map (Figures 1A,B). Compared with normal tissues, a total of 484 IRGs (173 up-regulated and 311 down-regulated) and 71 CRTFs (46 up-regulated and 25 down-regulated) were differentially expressed in CRC tissues. The heatmaps showed that CRC samples can be distinguished from normal samples based on the differentially expressed IRGs and CRTFs (Figures 1C,D). The volcano plots showed the distribution of differentially expressed IRGs and CRTF between CRC samples and normal controls (Figures 1E,F).

### Screening of IRGs Related to Significant Prognosis in CRC

**FIGURE 2:**
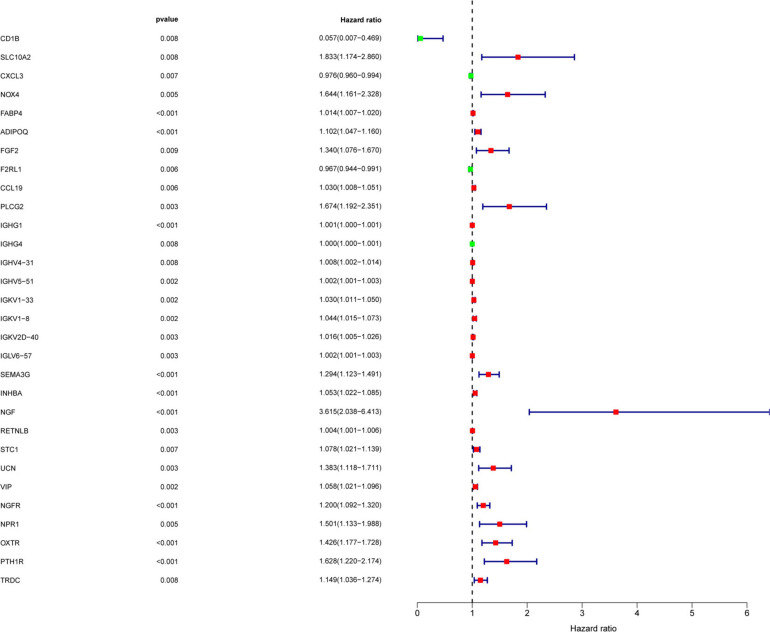
Screening of IRGs related to significant prognosis in CRC. Forest plot showing the prognostic immune-related genes, *P* values, and hazard ratios. Green dots represent low risk factors, and red dots represent high risk factors.

To determine the differentially expressed IRGs with prognostic characteristics, the relationship between the expression of 484 IRGs in 398 CRC samples and prognosis were evaluated by univariate Cox analysis. A total of 30 IRGs with prognostic characteristics were found, as shown in Table 1. Figure 2 is a forest plot showing the prognostic IRGs, *P* values, and hazard ratios. Among the 18 prognostic-related IRGs, CD1B, CXCL3, F2RL1, and IGHG4 are low-risk genes. The higher expression of these genes indicated better prognosis of patients. The other 14 IRGs are high-risk genes, and when their expression increases, the patient’s risk increases. NGF is the gene with the highest risk factor.

**TABLE 1:** General characteristics of prognostic immune-related genes.

Gene symbol	HR	HR.95L	HR.95H	*P*-value	Gene symbol	HR (95% CI)	*P*-value
CD1B	0.057	0.007	0.469	0.007658134	CD1B	0.057 (0.007–0.469)	0.008
SLC10A2	1.833	1.174	2.860	0.007661263	SLC10A2	1.833 (1.174–2.860)	0.008
CXCL3	0.976	0.960	0.994	0.007314764	CXCL3	0.976 (0.960–0.994)	0.007
NOX4	1.644	1.161	2.328	0.00512998	NOX4	1.644 (1.161–2.328)	0.005
FABP4	1.014	1.007	1.020	4.75E-05	FABP4	1.014 (1.007–1.020)	4.75E-05
ADIPOQ	1.102	1.047	1.160	0.00022247	ADIPOQ	1.102 (1.047–1.160)	2.22E-04
FGF2	1.340	1.076	1.670	0.009000359	FGF2	1.340 (1.076–1.670)	0.009
F2RL1	0.967	0.944	0.991	0.006461334	F2RL1	0.967 (0.944–0.991)	0.006
CCL19	1.030	1.008	1.051	0.005852187	CCL19	1.030 (1.008–1.051)	0.006
PLCG2	1.674	1.192	2.351	0.002941416	PLCG2	1.674 (1.192–2.351)	0.003
IGHG1	1.001	1.000	1.001	0.000824658	IGHG1	1.001 (1.000–1.001)	0.001
IGHG4	1.000	1.000	1.001	0.008255942	IGHG4	1.000 (1.000–1.001)	0.008
IGHV4-31	1.008	1.002	1.014	0.007667535	IGHV4-31	1.008 (1.002–1.014)	0.008
IGHV5-51	1.002	1.001	1.003	0.002062246	IGHV5-51	1.002 (1.001–1.003)	0.002
IGKV1-33	1.030	1.011	1.050	0.001978816	IGKV1-33	1.030 (1.011–1.050)	0.002
IGKV1-8	1.044	1.015	1.073	0.002316247	IGKV1-8	1.044 (1.015–1.073)	0.002
IGKV2D-40	1.016	1.005	1.026	0.003078753	IGKV2D-40	1.016 (1.005–1.026)	0.003
IGLV6-57	1.002	1.001	1.003	0.003346154	IGLV6-57	1.002 (1.001–1.003)	0.003
SEMA3G	1.294	1.123	1.491	0.000355359	SEMA3G	1.294 (1.123–1.491)	3.55E-04
INHBA	1.053	1.022	1.085	0.000678328	INHBA	1.053 (1.022–1.085)	0.001
NGF	3.615	2.038	6.413	1.12E-05	NGF	3.615 (2.038–6.413)	1.12E-05
RETNLB	1.004	1.001	1.006	0.002823016	RETNLB	1.004 (1.001–1.006)	0.003
STC1	1.078	1.021	1.139	0.007200166	STC1	1.078 (1.021–1.139)	0.007
UCN	1.383	1.118	1.711	0.002826168	UCN	1.383 (1.118–1.711)	0.003
VIP	1.058	1.021	1.096	0.001913177	VIP	1.058 (1.021–1.096)	0.002
NGFR	1.200	1.092	1.320	0.000160985	NGFR	1.200 (1.092–1.320)	1.61E-04
NPR1	1.501	1.133	1.988	0.004630474	NPR1	1.501 (1.133–1.988)	0.005
OXTR	1.426	1.177	1.728	0.000284786	OXTR	1.426 (1.177–1.728)	2.85E-04
PTH1R	1.628	1.220	2.174	0.000936214	PTH1R	1.628 (1.220–2.174)	0.001
TRDC	1.149	1.036	1.274	0.008408965	TRDC	1.149 (1.036–1.274)	0.008

### The Mechanism of Prognosis-Related IRGs and CRTF-IRG Regulatory Network

**FIGURE 3:**
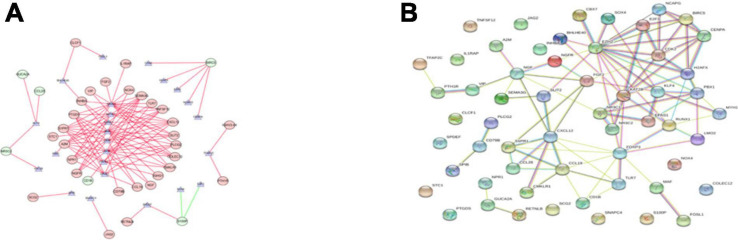
The main regulatory network constructed based on prognosis-related IRGs and CRTFs. **(A)** The main regulatory network was constructed using Cytoscape for visualization. The circulars represent differentially expressed prognostic immune-related genes, and the purple triangles represent prognosis-related cancer-related transcription factors, respectively. The red circulars represent high-risk genes, and the green circulars represent low-risk genes. Red lines represent positive correlations and green lines represent negative correlations. **(B)** The PPI network was predicted using the Search Tool for the Retrieval of Interacting Genes online database. Analyzing the functional interactions between proteins may provide insights into the mechanisms of generation or development of CRC.

We explored the potential regulatory mechanisms of 18 prognostic-related IRGs, which may reflect the regulatory mechanisms of these gene sets. We selected 30 prognostic-related IRGs and 71 differential CTRFs for correlation analysis to explore the regulatory mechanism of prognostic-related IRGs. The Cor. test function is used to test the correlation between each CRTF and each IRG. The core method is Pearson test. The correlation coefficient filter is 0.4, and the *P* value filter is 0.001. The regulatory relationship between these CRTFs and IRGs is revealed in the regulatory network (Figure 3A). As shown in Figure 3A, NR3C1, MYH11, RUNX1, MAF, CCB7, LMO2, FOXP3, and EPAS1 regulate most of the IRGs related to prognosis and dominate the regulation network. This transcriptional regulatory network reveals the regulatory relationship between these IRGs and CRTFs. Table 2 shows the correlation between IRGs and CRTFs after screening. The PPI network of IRGs and CRTFs was constructed, and the most significant module was obtained. The functional analyses of genes involved in this module were analyzed. Enrichment analysis shows that the genes in this module are mainly involved in cell proliferation and metabolic processes (Figure 3B).

**TABLE 2:** Correlation between prognostic IRGs and CRTFs.

TF	Immune Gene	Cor	*P* value	Regulation
BHLHE40	CLCF1	0.416839314	1.80E-15	Positive
BHLHE40	INHBA	0.401507385	2.28E-14	Positive
CBX7	CXCL12	0.486561766	2.97E-21	Positive
CBX7	PTGDS	0.503200159	7.70E-23	Positive
CBX7	COLEC12	0.424078295	5.19E-16	Positive
CBX7	A2M	0.616242787	2.60E-36	Positive
CBX7	CCL19	0.436562294	5.65E-17	Positive
CBX7	SEMA3G	0.542759756	5.56E-27	Positive
CBX7	SLIT2	0.555854437	1.78E-28	Positive
CBX7	TNFSF12	0.563264084	2.36E-29	Positive
CBX7	NGFR	0.488603311	1.92E-21	Positive
CBX7	NPR1	0.531260451	1.01E-25	Positive
CBX7	S1PR1	0.592504631	4.92E-33	Positive
CDK2	BIRC5	0.431405235	1.43E-16	Positive
CENPA	BIRC5	0.642177664	3.16E-40	Positive
E2F3	S100P	−0.44339107	1.61E-17	Negative
EPAS1	CXCL12	0.453700715	2.31E-18	Positive
EPAS1	PTGDS	0.448012905	6.81E-18	Positive
EPAS1	A2M	0.55619075	1.62E-28	Positive
EPAS1	CCL19	0.413149974	3.35E-15	Positive
EPAS1	PLCG2	0.446388336	9.24E-18	Positive
EPAS1	SEMA3G	0.484129321	4.99E-21	Positive
EPAS1	NPR1	0.424577277	4.75E-16	Positive
EPAS1	S1PR1	0.562256083	3.12E-29	Positive
EZH2	BIRC5	0.404514518	1.40E-14	Positive
FOSL1	CLCF1	0.539965172	1.14E-26	Positive
FOXP3	CD1B	0.512515443	9.11E-24	Positive
FOXP3	PTGDS	0.427576845	2.81E-16	Positive
FOXP3	A2M	0.545665937	2.62E-27	Positive
FOXP3	TLR7	0.505943161	4.13E-23	Positive
FOXP3	PLCG2	0.444809183	1.24E-17	Positive
FOXP3	IGHG1	0.445339174	1.12E-17	Positive
FOXP3	CMKLR1	0.65044552	1.48E-41	Positive
FOXP3	TNFSF12	0.462021403	4.58E-19	Positive
FOXP3	S1PR1	0.491569563	1.01E-21	Positive
H2AFX	BIRC5	0.459290301	7.83E-19	Positive
KAT2B	NR3C2	0.453792752	2.27E-18	Positive
KLF4	CCL28	0.428000208	2.61E-16	Positive
KLF4	GUCA2A	0.437937735	4.40E-17	Positive
KLF4	NR3C2	0.565658867	1.22E-29	Positive
LMO2	CXCL12	0.574574237	9.84E-31	Positive
LMO2	PTGDS	0.539133308	1.40E-26	Positive
LMO2	COLEC12	0.410459525	5.26E-15	Positive
LMO2	A2M	0.589918106	1.08E-32	Positive
LMO2	CCL19	0.452942043	2.67E-18	Positive
LMO2	CD79B	0.43546919	6.88E-17	Positive
LMO2	PLCG2	0.496304811	3.59E-22	Positive
LMO2	SEMA3G	0.575029816	8.63E-31	Positive
LMO2	SLIT2	0.461147564	5.44E-19	Positive
LMO2	CMKLR1	0.465054783	2.51E-19	Positive
LMO2	NGF	0.408695876	7.04E-15	Positive
LMO2	TNFSF12	0.497580181	2.70E-22	Positive
LMO2	NGFR	0.456316187	1.40E-18	Positive
LMO2	NPR1	0.546869058	1.92E-27	Positive
LMO2	S1PR1	0.635395146	3.63E-39	Positive
MAF	CXCL12	0.660448884	3.22E-43	Positive
MAF	PTGDS	0.524781296	4.95E-25	Positive
MAF	COLEC12	0.700804251	1.23E-50	Positive
MAF	A2M	0.72079867	8.62E-55	Positive
MAF	NOX4	0.609211362	2.60E-35	Positive
MAF	TLR7	0.658440876	7.02E-43	Positive
MAF	PLCG2	0.507215446	3.09E-23	Positive
MAF	IGHG1	0.437003034	5.21E-17	Positive
MAF	SEMA3G	0.581610292	1.28E-31	Positive
MAF	SLIT2	0.621276631	4.82E-37	Positive
MAF	CMKLR1	0.683628325	2.48E-47	Positive
MAF	INHBA	0.59794424	9.22E-34	Positive
MAF	NGF	0.430176879	1.78E-16	Positive
MAF	STC1	0.438639668	3.87E-17	Positive
MAF	TNFSF12	0.632265446	1.10E-38	Positive
MAF	NGFR	0.417731411	1.55E-15	Positive
MAF	NPR1	0.590835707	8.17E-33	Positive
MAF	S1PR1	0.714778352	1.68E-53	Positive
MYH11	CXCL12	0.40925357	6.42E-15	Positive
MYH11	PTGDS	0.422386792	6.95E-16	Positive
MYH11	A2M	0.613726369	5.96E-36	Positive
MYH11	SEMA3G	0.427612739	2.79E-16	Positive
MYH11	SLIT2	0.555833348	1.79E-28	Positive
MYH11	TNFSF12	0.461576107	5.00E-19	Positive
MYH11	VIP	0.470463748	8.48E-20	Positive
MYH11	NGFR	0.456187973	1.43E-18	Positive
MYH11	NPR1	0.520453508	1.40E-24	Positive
MYH11	S1PR1	0.590045306	1.04E-32	Positive
NCAPG	BIRC5	0.554839718	2.33E-28	Positive
NR3C1	CXCL12	0.589098923	1.38E-32	Positive
NR3C1	PTGDS	0.449503703	5.14E-18	Positive
NR3C1	COLEC12	0.62546825	1.16E-37	Positive
NR3C1	A2M	0.675323103	8.15E-46	Positive
NR3C1	NOX4	0.502386799	9.25E-23	Positive
NR3C1	TLR7	0.549000706	1.10E-27	Positive
NR3C1	FGF2	0.456714287	1.29E-18	Positive
NR3C1	PLCG2	0.465257317	2.41E-19	Positive
NR3C1	SEMA3G	0.512080143	1.01E-23	Positive
NR3C1	SLIT2	0.584275041	5.83E-32	Positive
NR3C1	CMKLR1	0.547817016	1.50E-27	Positive
NR3C1	INHBA	0.559600609	6.44E-29	Positive
NR3C1	TNFSF12	0.482380673	7.22E-21	Positive
NR3C1	VIP	0.420746246	9.23E-16	Positive
NR3C1	IL1RAP	0.409789576	5.88E-15	Positive
NR3C1	NPR1	0.46851997	1.26E-19	Positive
NR3C1	S1PR1	0.646179039	7.28E-41	Positive
PBX1	A2M	0.405034889	1.28E-14	Positive
RUNX1	CXCL12	0.438209268	4.19E-17	Positive
RUNX1	COLEC12	0.407539671	8.52E-15	Positive
RUNX1	A2M	0.433301718	1.02E-16	Positive
RUNX1	SEMA3G	0.416583417	1.88E-15	Positive
RUNX1	INHBA	0.435841724	6.44E-17	Positive
RUNX1	S1PR1	0.40202312	2.10E-14	Positive
SNAPC4	JAG2	0.402211118	2.03E-14	Positive
SOX4	S100P	−0.415710928	2.18E-15	Negative
SPDEF	S100P	0.425063976	4.37E-16	Positive
SPDEF	RETNLB	0.495866186	3.95E-22	Positive
SPIB	SCG2	0.419892177	1.07E-15	Positive
TFAP2C	IGHV3-64	0.507706392	2.76E-23	Positive
TFAP2C	PTH1R	0.459218282	7.94E-19	Positive

### Hub Gene Selection and Analysis in CRC

**FIGURE 4:**
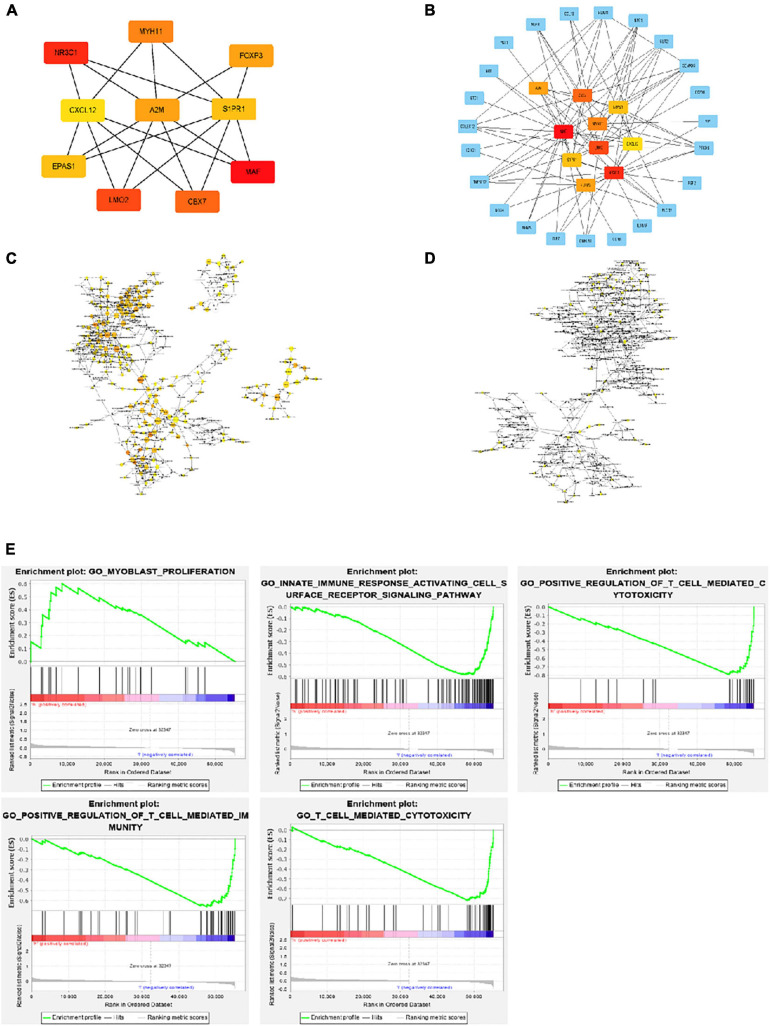
Interaction network and biological process analysis of the hub genes. **(A)** Top 10 hub genes screened from the regulatory network. **(B)** Top 10 hub genes and their first neighbors that are screened from the regulatory network. Hub gene is shown in red to orange on the left. The first neighboring node is shown in blue. The right picture shows the characteristics of the genes in the left picture. The green ones are low-risk genes. The red ones are high-risk genes. The triangles are transcription factors. **(C)** The biological process analysis of genes in the network was constructed using BiNGO. The color depth of nodes refers to the corrected *P* value of ontologies. The size of nodes refers to the number of genes that are involved in the ontologies. *P* < 0.01 was considered statistically significant. **(D)** The biological process analysis of hub genes was constructed using BiNGO. *P* < 0.05 was considered statistically significant. **(E)** Ten-hub-gene enrichment plots from Gene Set Enrichment Analysis (GSEA).

Using Cytohubba in Cytoscape, we filtered 33 hub genes that were identified by filtering according to the criterion of degrees > 10 criteria (each node had more than 10 interactions), and the 10 most central genes in the immune gene regulatory network according to node degree were MAF, A2M, CBX7, MYH11, EPAS1, CXCL12, LMO2, S1PR1, FOXP3, and NR3C1 (Figures 4A,B). Gene Ontology (GO) enrichment analysis of genes in the immune gene regulatory network related to prognosis was conducted to explore which signaling pathways were activated. The analysis of the biological processes (BPs) of the central genes using BiNGO in Cytoscape is shown in Figures 4C,D. GO analysis showed that the changes in the BPs of these genes were significantly enriched in the immune process, cell proliferation, immune organ development, and hemopoiesis. Changes in molecular function were mainly focused on TF activity, cytokine activation, and molecular binding. Afterward, the functional enrichment analysis of the key genes of the IRG set was performed by GSEA. Figure 4E shows that the changes in the BP of these genes are significantly enriched in the immune process, cell proliferation, immune organ development, and hematopoiesis. The results of Kaplan–Meier analysis of these hub genes are in the Supplementary Figure 1.

### Construction of the Immune-Related Signature for CRC

**FIGURE 5:**
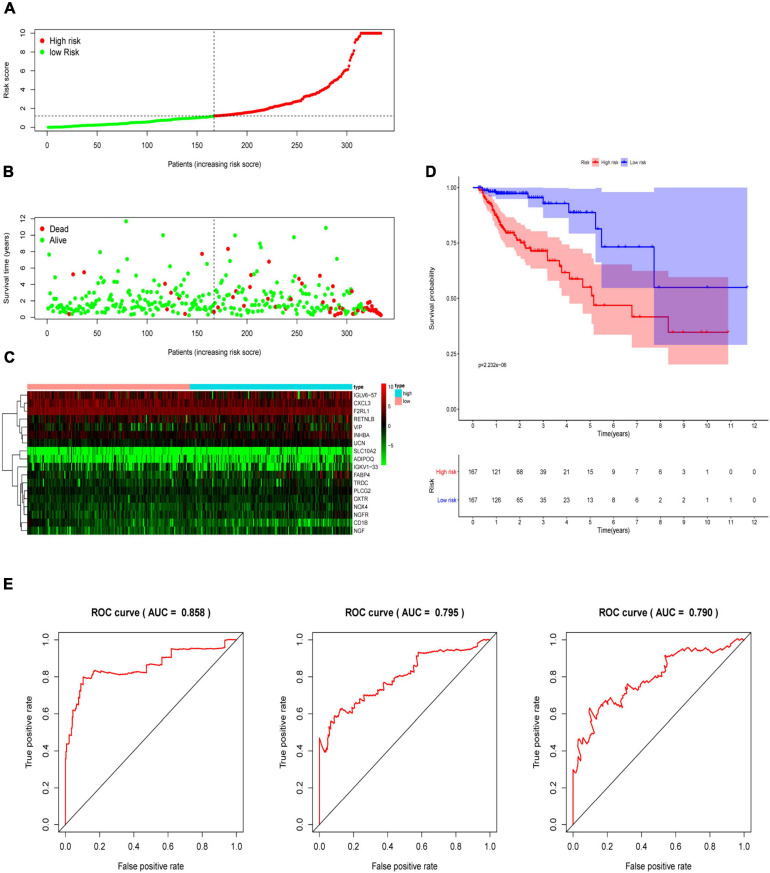
Construction of an immune-related prognostic signature for CRC. **(A)** The risk score distribution of CRC patients in The Cancer Genome Atlas (TCGA) database. **(B)** Survival status and duration of patients. **(C)** Heatmap of the expression of 18 immune-related genes in CRC patients. **(D)** Survival curves for the low-risk and high-risk groups. **(E)** The receiver operating characteristic curve (ROC) analysis predicted overall survival using the risk score. The forecast time is 1, 3, and 5 years.

Multivariate Cox analysis was performed on 30 prognostic IRGs, and 18 genes were finally selected to establish a prognostic model (Table 3). The risk score is based on the gene expression level multiplied by its corresponding regression coefficient. The regression coefficient was calculated by multivariate Cox regression. The risk score is related to not only the expression level of these genes but also the correlation coefficients. The risk score of each patient is the sum of all the 18 risk prognostic genes in Table 3 multiplied by the corresponding risk factors. The 398 CRC samples were then divided into high-risk groups (*n* = 199) and low-risk groups (*n* = 199) based on the median risk score (Figure 5A). Survival overview and gene expression heatmaps are presented in Figures 5B,C. Survival analysis showed that the OS of patients in the high-risk group was significantly lower than that in the low-risk group (*P* < 0.0001; Figure 5D). The 5-year survival rate of the high-risk group was 51.1%, and the 5-year survival rate of the low-risk group was 81.4%. The areas under the ROC curves at 1, 3, and 5 years of OS are 0.811, 0.711, and 0.734, respectively, which indicated that the prognostic model showed good sensitivity and specificity (Figure 5E). In addition, as shown in Supplementary Figure 2, the model after excluding genes with *P* ≥ 0.05 has advantages in the short-term prognosis (1 year), but the model is not effective in predicting the long-term prognosis.

**TABLE 3:** Eighteen genes that constitute the immune-related prognostic model and the corresponding risk factors Riskscore = CD1B*(-4.726) + SLC10A2*(0.844) + CXCL3*(-0.019) + NOX4*(-1.253) + FABP4*(0.057) + ADIPOQ*(-0.249) + F2RL1*(-0.027) + PLCG2*(0.499) + IGKV1 - 33*(0.051) + IGLV6 - 57*(0.003) + INHBA*(0.139) + NGF*(0.944) + RETNLB*(0.004) + UCN*(0.468) + VIP*(0.067) + NGFR*(-0.436) + OXTR*(-0.304) + TRDC*(0.267).

Gene symbol	Coef	HR	HR.95L	HR.95H	*P* value	Gene symbol	Coef	HR (95% CI)	*P*-value
CD1B	−4.72557	0.008866	0.000607	0.12956	0.000554	CD1B	−4.726	0.009 (0.001–0.130)	0.001
SLC10A2	0.844378	2.326529	1.393175	3.885182	0.00125	SLC10A2	0.844	2.327 (1.393–3.885)	0.001
CXCL3	−0.01882	0.981352	0.962769	1.000294	0.053627	CXCL3	−0.019	0.981 (0.963–1.000)	0.054
NOX4	−1.25348	0.28551	0.077017	1.058413	0.060787	NOX4	−1.253	0.286 (0.077–1.058)	0.061
FABP4	0.056939	1.058591	1.017112	1.101762	0.00524	FABP4	0.057	1.059 (1.017–1.102)	0.005
ADIPOQ	−0.24929	0.779353	0.615716	0.986479	0.038156	ADIPOQ	−0.249	0.779 (0.616–0.986)	0.038
F2RL1	−0.02671	0.97364	0.947442	1.000561	0.054904	F2RL1	−0.027	0.974 (0.947–1.001)	0.055
PLCG2	0.499377	1.647695	1.049015	2.588045	0.030183	PLCG2	0.499	1.648 (1.049–2.588)	0.030
IGKV1-33	0.051157	1.052488	1.013034	1.093479	0.008684	IGKV1-33	0.051	1.052 (1.013–1.093)	0.009
IGLV6-57	0.002935	1.002939	1.001397	1.004484	0.000186	IGLV6-57	0.003	1.003 (1.001–1.004)	<0.001
INHBA	0.139399	1.149582	1.032699	1.279695	0.010831	INHBA	0.139	1.150 (1.033–1.280)	0.011
NGF	0.943896	2.569974	0.919885	7.179992	0.071757	NGF	0.944	2.570 (0.920–7.180)	0.072
RETNLB	0.004124	1.004132	1.001374	1.006897	0.003296	RETNLB	0.004	1.004 (1.001–1.007)	0.003
UCN	0.468088	1.596938	1.251116	2.038348	0.00017	UCN	0.468	1.597 (1.251–2.038)	<0.001
VIP	0.066515	1.068777	1.004482	1.137187	0.035621	VIP	0.067	1.069 (1.004–1.137)	0.036
NGFR	−0.43637	0.646376	0.412695	1.012376	0.05662	NGFR	−0.436	0.646 (0.413–1.012)	0.057
OXTR	−0.30443	0.737541	0.524661	1.036796	0.079773	OXTR	−0.304	0.738 (0.525–1.037)	0.080
TRDC	0.267054	1.306111	1.14942	1.484163	4.21E-05	TRDC	0.267	1.306 (1.149–1.484)	<0.001

### Immune-Related Prognostic Signature Was an Independent Predictive Marker of OS for CRC Patients

**FIGURE 6:**
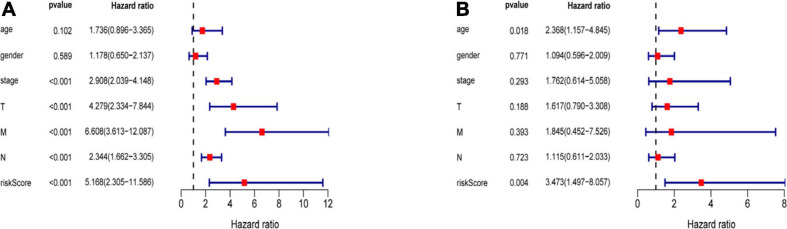
Independence of immune-related prognostic signature from clinical factors. **(A)** Forest plot for univariate analysis of overall survival of TCGA CRC patients. **(B)** Forest plot for multivariate analysis of overall survival of TCGA CRC patients. Red dots represent high-risk factors.

Three hundred ninety-eight CRC patients with clinical information of age, gender, pathological stage, TNM stage, and risk score were selected for further analysis. Univariate and multivariate Cox regression analyses were performed to assess the independent predictive power of immune-related prognostic markers. Univariate analysis showed that pathological stage (*P* < 0.001), TNM stage (*P* < 0.001), and immune-related prognostic risk score (*P* < 0.001) were significantly correlated with OS (Table 4 and Figure 6A). After multivariate analysis, the immune-related prognostic risk score was the only independent prognostic factor related to OS (*P* < 0.005; Table 5 and Figure 6B).

**TABLE 4:** Univariate analyses of overall survival in CRC patients of TCGA.

Variable	HR	HR.95L	HR.95H	*P* value	Variable	HR	*P*-value
Age	1.736	0.896	3.365	0.102	Age	1.736 (0.896–3.365)	0.102
Gender	1.178	0.65	2.137	0.589	Gender	1.178 (0.650–2.137)	0.589
Stage	2.908	2.039	4.148	<0.001	Stage	2.908 (2.039–4.148)	<0.001
*T*	4.279	2.334	7.844	<0.001	*T*	4.279 (2.334–7.844)	<0.001
*M*	6.608	3.613	12.087	<0.001	*M*	6.608 (3.613–12.087)	<0.001
*N*	2.344	1.662	3.305	<0.001	*N*	2.344 (1.662–3.305)	<0.001
riskScore	5.168	2.305	11.586	<0.001	riskScore	5.168 (2.305–11.586)	<0.001

**TABLE 5:** Multivariate analyses of overall survival in CRC patients of TCGA.

Variables	HR	HR.95L	HR.95H	*P* value	Variables	HR (95% CI)	*P*-value
Age	2.367721	1.157118	4.844884	0.018303	Age	2.368 (1.157–4.845)	0.018
Gender	1.094256	0.595933	2.009281	0.771426	Gender	1.094 (0.596–2.009)	0.771
Stage	1.761849	0.613679	5.058204	0.292554	Stage	1.762 (0.614–5.058)	0.293
*T*	1.616913	0.790257	3.308302	0.188337	*T*	1.617 (0.790–3.308)	0.188
*M*	1.84477	0.452177	7.526206	0.393327	*M*	1.845 (0.452–7.526)	0.393
*N*	1.114703	0.611102	2.033314	0.723281	*N*	1.115 (0.611–2.033)	0.723
riskScore	3.472938	1.497053	8.056694	0.003734	riskScore	3.473 (1.497–8.057)	0.004

### Association Between 18 IRGs, Clinical Parameters, and Prognostic Risk Scores

**FIGURE 7:**
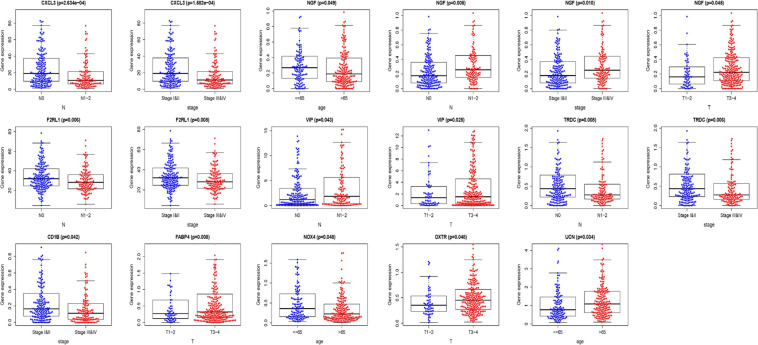
Clinical characteristics correlation analysis. Clinical characteristics correlation analysis of genes in the risk score model (*P* < 0.05).

We analyzed the association between the expression of 18 key prognostic related IRGs in the patient’s tumor tissue and the patient’s clinical characteristics. The association between NGF, TRDC, CXCL3, CD1B, VIP, F2RL1, FABP4, OXTR, UCN, NOX4, ADIPOQ, and clinical characteristics was found (Table 6 and Figure 7). NGF is negatively correlated with age, and NGF expression is generally higher in advanced patients. Patients with higher VIP expression generally have higher T and N stages. On the other hand, TRDC, CXCL3, and FRL1 are highly expressed in patients in the early stage and patients with N0 stage.

**TABLE 6:** Eighteen genes in the risk score model and clinical characteristics correlation analysis.

Gene symbol	Age	Gender	Stage	*T*	*M*	*N*
CD1B	−0.983 (0.326)	1.524 (*A*200.129)	2.047 (0.042)	1.837 (0.069)	17.361 (5.956e−04)	1.883 (0.061)
SLC10A2	0.57 (0.569)	1.211 (0.228)	0.673 (0.501)	−0.577 (0.565)	2.255 (0.521)	0.63 (0.529)
CXCL3	−0.593 (0.554)	−0.845 (0.399)	3.828 (1.582*e*−04)	0.582 (0.562)	8.458 (0.037)	3.696 (2.634e−04)
NOX4	1.983 (0.048)	−0.889 (0.375)	−0.739 (0.460)	−0.221 (0.826)	1.356 (0.716)	−1.146 (0.253)
FABP4	1.665 (0.098)	0.985 (0.326)	−0.97 (0.333)	−2.659 (0.008)	3.322 (0.345)	−1.062 (0.290)
ADIPOQ	1.49 (0.138)	0.232 (0.817)	−0.467 (0.641)	−2.356 (0.019)	1.274 (0.735)	−0.578 (0.564)
F2RL1	−1.259 (0.209)	0.132 (0.895)	2.675 (0.008)	0.839 (0.404)	1.936 (0.586)	2.752 (0.006)
PLCG2	−0.344 (0.731)	0.971 (0.333)	−1.347 (0.179)	−0.604 (0.547)	2.094 (0.553)	−1.693 (0.092)
IGKV1-33	−0.892 (0.373)	−1.171 (0.243)	1.102 (0.272)	−0.602 (0.548)	5.168 (0.160)	1.094 (0.275)
IGLV6-57	−0.47 (0.639)	−0.851 (0.396)	−0.451 (0.653)	0.023 (0.982)	3.862 (0.277)	−0.532 (0.596)
INHBA	1.674 (0.095)	−0.644 (0.520)	−0.861 (0.390)	−0.854 (0.396)	1.453 (0.693)	−1.192 (0.234)
NGF	1.982 (0.049)	0.97 (0.333)	−2.586 (0.010)	−2.025 (0.045)	3.632 (0.304)	−2.864 (0.005)
RETNLB	0.556 (0.579)	0.35 (0.727)	1.355 (0.176)	1.381 (0.172)	1.72 (0.633)	1.273 (0.204)
UCN	−2.129 (0.034)	1.217 (0.225)	−1.575 (0.117)	−0.26 (0.795)	0.735 (0.865)	−1.428 (0.155)
VIP	0.486 (0.627)	−0.139 (0.889)	−1.763 (0.080)	−2.259 (0.025)	0.968 (0.809)	−2.041 (0.043)
NGFR	1.548 (0.124)	1.651 (0.100)	−1.511 (0.133)	−1.652 (0.101)	4.464 (0.216)	−1.626 (0.106)
OXTR	0.297 (0.767)	−1.763 (0.080)	−1.243 (0.215)	−1.985 (0.048)	1.805 (0.614)	−1.272 (0.205)
TRDC	0.144 (0.885)	−0.141 (0.888)	2.772 (0.006)	1.011 (0.316)	14.881 (0.002)	2.671 (0.008)
riskScore	−1.146 (0.253)	−0.901 (0.369)	−1.268 (0.207)	−1.394 (0.165)	17.773 (4.899*e*−04)	−1.274 (0.205)

*The numbers in the table represent the *t* value of *t* test between each gene and clinical features; the numbers in parentheses represent *P* value.*

### TIMER Database Analysis

**FIGURE 8:**
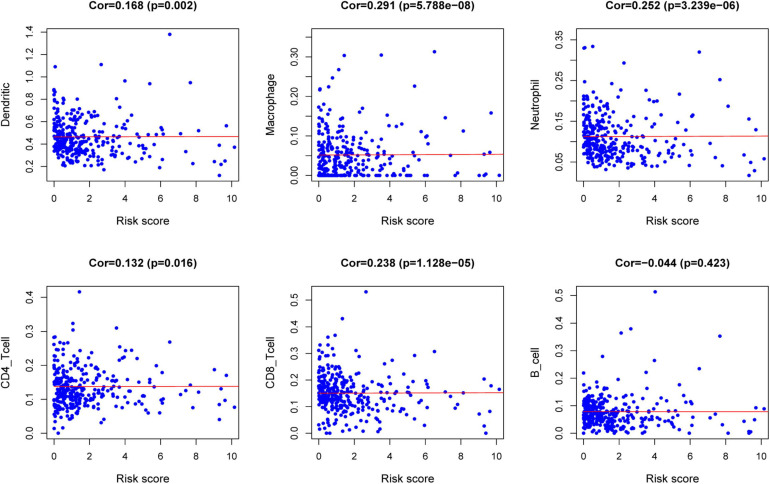
Relationships between the risk score model and infiltration abundances of six types of immune cells.

The relationships between the risk score model and immune cell infiltration were studied. The characterization of immune infiltration is very important for exploring the state of the immune microenvironment and studying the interaction between tumors and immunity. We applied the TIMER tool to identify potential relationships between IPS and infiltrating immune cells, including B cells, CD4^+^ T cells, CD8^+^ T cells, neutrophils, macrophages, and dendritic cells. As shown in Figure 8, the proportions of tumor-infiltrating CD4^+^ T cells, CD8^+^ T cells, neutrophils, macrophages, and dendritic cells were closely related to our prognostic risk score (*p* < 0.05).

## Discussion

In recent years, the genetic characteristics of mRNA in cancer patients have attracted people’s attention, and studies have revealed its great potential in the prognosis of CRC. In this study, based on the analysis of the TCGA data set, 484 differentially expressed IRGs were screened from 389 HCC and 39 normal tissues. By univariate regression analysis of differentially expressed IRGs, 30 genes were detected to be significantly correlated with OS. To further study the regulatory mechanisms of prognostic IRGs, a tumor-related TF-mediated network was established to reveal key TFs that can regulate these IRGs. Studies have shown that CBX7 played an important role in gastric and pancreatic cancer (Ni et al., 2017, 2018). In recent years, studies have found that CBX7 was a component of polycomb repressive complex 1, maintaining the stem cell–like characteristics of gastric cancer cells by activating the AKT pathway and down-regulating p16 (Ni et al., 2018). MYH11 (also known as SMMHC) encodes a smooth muscle myosin heavy chain, which plays a key role in smooth muscle contraction. The inversion of the MYH11 locus is one of the most common chromosomal aberrations in acute myeloid leukemia (Alhopuro et al., 2008). The MYH11 gene has a single-nucleotide repeat sequence (C8) in the coding sequence, which may be a mutation target for cancer that exhibits microsatellite instability (MSI). The study found that compared with the low microsatellite unstable group, the incidence of MYH11 frameshift mutation was higher in patients with high microsatellite-unstable (MSI) gastric cancer and CRC (Jo et al., 2018). Among these major hub genes, the study of CXCL12 is more comprehensive. It has been reported that the CXCL12/CXCR4 axis is related to tumor progression, angiogenesis, metastasis, and survival (Teicher and Fricker, 2010). Recent studies have found that the activation of LMO2 is essential for the development of T-cell acute lymphoblastic leukemia (T-ALL) leukemia (Morishima et al., 2019). The SP1PR1 gene plays a role in regulating tumors. Targeting the SphK1/S1P/S1PR1 axis with specific drugs can reduce tumor progression caused by key proinflammatory cytokines, macrophage infiltration, and obesity (Nagahashi et al., 2018). FOXP3 is one of the key TFs controlling the development and function of regulatory T cells. FOXP3 has been extensively studied in human tumors, which is closely related to tumor immunity, and its correlation with T cells in tumors has recently been reported (Wing et al., 2019). The relationship between several other genes and CRC is still unclear. Among them, the role of MAF, A2M, EPAS1, and NR3C1 in CRC is worthy of further investigation. A study of breast cancer showed that the enhanced expression of MAF can mediate bone metastasis of breast cancer, which can be used as a risk index for bone metastasis in breast cancer patients (Pavlovic et al., 2015). The proteins encoded by A2M are protease inhibitors and cytokine transporters. It can inhibit a variety of proteases, as well as inflammatory cytokines, thereby destroying the inflammatory cascade. Xu’s team found that *EPAS1* gene is dysregulated in non–small cell lung cancer, which encodes hypoxia-inducible factor 2α and plays an important role in the progression of non–small cell lung cancer (Xu et al., 2018). It is known that EPAS1 is regulated by DNA methylation transcription in CRC (Rawluszko-Wieczorek et al., 2014), but its role in CRC remains to be studied. NR3C1 encodes a glucocorticoid receptor, which can act both as a TF that binds to the glucocorticoid response element in the promoter of the glucocorticoid response gene to activate its transcription and as a regulator of other TFs. Further experimental evidence on the function of these genes in CRC may be of great help to our understanding of the progress of CRC.

In recent years, Xie et al. (2017) established a 20-gene prognosis model, which has a good predictive function for CRC prognosis. Another study also constructed a novel four-gene signature for CRC OS prediction based on gene expression data from TCGA, COAD, and READ data sets (Ahluwalia et al., 2019). A recent study exploring the prognostic value of immune cells in the CRC tumor microenvironment determined that tumor-infiltrating immune cells is highly correlated with the progression and prognosis of CRC (Ge et al., 2019). However, these studies do not fully explore the relationship between immune genes and the prognosis of CRC. Our study has the following advantages. First, we used a specialized immunological database to analyze as many IRGs as possible. To our knowledge, this is the first study to explore the relationship between a large number of IRGs and the prognosis of patients with CRC. Second, we obtained some immune-related prognostic genes and established a novel prognostic model related to immunity. This prognostic model showed excellent performance in the prediction of OS based on the TCGA database. According to the in-depth analysis, the immune-related prognostic model was demonstrated to be an independent prognostic indicator after adjusting for other clinical factors. These results indicated that the immune-related prognosis model can be used as an effective marker for the prognosis of CRC patients.

The characterization of immune infiltration is of great significance for studying the interaction between tumor and immunity. Therefore, we explored the relationship between immune-related prognostic models and immune cell infiltration to reflect the state of the immune microenvironment. According to the TIMER database, we found that high-risk patients had higher levels of CD4^+^ T cells, CD8^+^ T cells, neutrophils, macrophages, and dendritic cells of infiltration. These results confirmed and extended the discovery that the heterogeneity of immune infiltration is important for the progression of CRC. A recent study reported that the colonic cancer microenvironment uses dendritic cells’ plasticity to support cancer progression by enhancing the release of the inflammatory chemokine CXCL1 (Hsu et al., 2018), which is consistent with our results. Neutrophils contribute to the activation, regulation, and effect of immune cells (Mantovani et al., 2011). Existing research reported that tumor-associated neutrophils in CRC produce matrix metalloproteinase 9 vascular endothelial growth factor and hepatocyte growth factor to promote tumor invasion and angiogenesis. In addition, neutrophils also promote the spread of tumor cells by capturing tumor cells in the circulation, thereby promoting their migration to distant places (Mizuno et al., 2019). Studies have reported that macrophages are associated with CRC progression (Wei et al., 2019). Tumor-associated macrophages (TAMs) can induce EMT processes to enhance CRC migration, invasion, and circulating tumor cell (CTC)-mediated metastasis (Wei et al., 2019). The immune model can indicate the infiltration of immune cells to some extent. It may be a promising way to cure CRC by broadening the relationship between immune cells and tumor progression.

Current research provides novel insights into the CRC immune microenvironment and immunotherapy. We conducted functional studies on selected genes to confirm their clinical value. However, the limitation of this study is that it is a retrospective study. Therefore, further prospective research is needed. On the one hand, the predictive capability of this model in CRC requires further testing with the goal of better prognostic stratification and treatment management. On the other hand, we need to further study the biological functions of the 18 IRGs through a series of experiments.

In short, through comprehensive analysis, many IRGs were found to be significantly related to the prognosis of CRC. Besides, we constructed a novel immune-related prognosis model as an independent prognostic indicator of CRC. This prognostic model can also indicate the infiltration of immune cells and prove its key role in the TIME. The current research has deepened our understanding of IRGs in CRC and provided new potential prognostic and therapeutic biomarkers.

## Data Availability Statement

The original contributions presented in the study are included in the article/Supplementary Material, further inquiries can be directed to the corresponding author.

## Author Contributions

YQ conceived the research. YQ and JW designed the research process, conducted bioinformatics analysis, downloaded and collated the data in the article, and wrote the first draft of the article. YQ, JW, and WL performed statistical analysis on the data. WL revised the article strictly to obtain the necessary knowledge and administrative support. FS, MH, KJ, DF, XZ, XK, QX, YH, and KD reviewed and edited the manuscript. All authors read and approved the final manuscript.

## Conflict of Interest

The authors declare that the research was conducted in the absence of any commercial or financial relationships that could be construed as a potential conflict of interest.

## Footnotes

1 https://portal.gdc.cancer.gov/

2 https://immport.niaid.nih.gov

3 http://cistrome.org/CistromeCancer/CancerTarget/

4 http://string-db.org, version 11.0

5 https://pypi.org/project/gseapy/

6 https://cistrome.shinyapps.io/timer/

## Supplementary Material

Supplementary Figure 1 The results of Kaplan–Meier analysis of 10 hub genes.

Supplementary Figure 2 Construction of an immune-related prognostic signature for CRC. **(A)** The risk score distribution of CRC patients in The Cancer Genome Atlas (TCGA) database. **(B)** Survival status and duration of patients. **(C)** Heatmap of the expression of 12 immune-related genes in CRC patients. **(D)** Survival curves for the low-risk and high-risk groups. **(E)** The receiver operating characteristic curve (ROC) analysis predicted overall survival using the risk score. The forecast time is 1, 3, and 5 years.
