# Supplementary figures and images for: Prognostic Risk Model of Immune-Related Genes in Colorectal Cancer
Source: Front Genet. 2021 Mar 4;12:619611. doi: 10.3389/fgene.2021.619611 (PMC7970128; doi:10.3389/fgene.2021.619611)

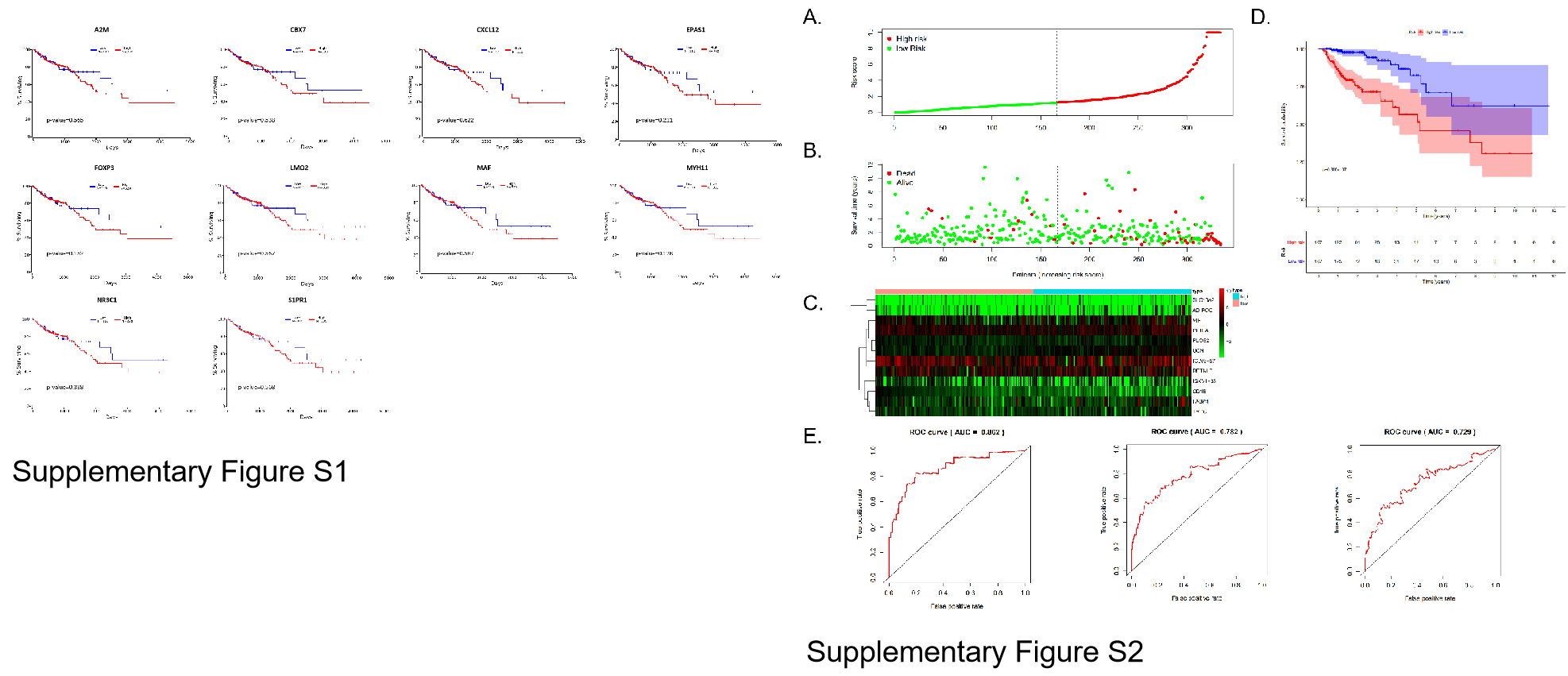

Supplement: Supplementary Figure 1 — The results of Kaplan–Meier analysis of 10 hub genes. [file Image_1.TIF]
